# Dataset in the production of composite clay-zeolite membranes made from naturally occurring clay minerals

**DOI:** 10.1016/j.dib.2018.06.117

**Published:** 2018-07-06

**Authors:** Abdelaziz Elgamouz, Najib Tijani

**Affiliations:** a Department of Chemistry, College of Sciences, University of Sharjah, P.O. Box 27272, Sharjah, United Arab Emirates; b Group: Membranes, Matériaux et Procédés de Séparation, Faculté des Sciences, Université Moulay Ismaîl, Meknès, Morocco

## Abstract

The data presented in this article are generated as part of the research article entitled “from a naturally occurring material (clay mineral) to the production of porous ceramic membranes” (Elgamouz and Tijani, 2018) [1]. This article describe how clays as very abundant versatile materials that have many properties not available in pure materials namely, silica, alumina and zirconia can be used for the preparation of ceramic membranes (Karaborni et al., 1996; Oun et al., 2017; Hollanders et al., 2016; de Oliveira Henriques et al., 2017) [2], [3], [4], [5]. This paper presents data obtained at different stages of the fabrication of a clay-zeolite composite ceramic membrane made from a largely available clay from the central region of Morocco (Meknes). The data include the characterization of the clay powder using XRD, FTIR, thermogravimetric (TGA and TDA) analysis of the clay powder. The data of porosity, mesoporosity, specific surface area, volumes of the pores, volumes of mesopores, diameters of the pores using mercury intrusion porosimetry and adsorption desorption of nitrogen data that was computed from BET and BJH theories of the clay supports at different firing temperatures (700, 750, 800, 850 and 900 °C). Data obtained from measurement of nitrogen permeation of support alone and that of the silicalite membranes are also represented.

**Specifications Table**

**Table:** 

Subject area	*chemistry*
More specific subject area	*Separation science/membranes*
Type of data	Table, image, text file, graph, figure
How data was acquired	ARL-8660 X-Ray Fluorescence Spectrometer
	SETARAM TGA instrument
	Bruker Platinum ATR tensor II FTIR spectrometer
	NABER 2804 furnace
	ASAP2010 Micrometrics apparatus was used to characterize the pore structures and the specific surface area of the supports.
	GC VARIAM model GC-4900 was hyphened to an apparatus designed specifically for these studies.
	Membranes were characterized by *X-ray diffraction* (*XRD*) using a D-Max Rigaku X-ray diffractometer with a copper anode and a graphite monochromator to select CuK_α1_ radiation (*λ* = 1.540 Å).
Data format	Raw, filtered, analyzed
Experimental factors	Natural clay was grinded into particles with size in the range of 250–315 µm using sieves standardized according to AFNOR. Flat-disk supports were obtained by uniaxial pressure.
Experimental features	The membranes were characterized and later used for the filtration of three gases: nitrogen (N_2_), sulfur hexafluoride SF_6_ and propane (C_3_).
Data source location	Mekenes, Morocco, The Latitude and Longitude of Meknes-El Menzeh is 33.9025 and − 5.5341 respectively. (DMS Coordinates 33°58′39.40′′ N -5°31′29.86′′ W) for collected clay samples.
Data accessibility	The data represented is with this article.
Related research article	This data article is submitted as a companion paper to the research article entitled: “From a naturally occurring material (clay mineral) to the production of porous ceramic membranes”, by A. Elgamouz, N. Tijani, published in the Journal of Microporous and Mesoporous Materials. [1]

**Value of the data**

• The data presents very useful results for the applications of clay-zeolite composite ceramic membranes in gas filtration.
• This data gives a detailed and complete set of experiments on the characterization of the porosity of clay-zeolite composite membranes that could be relied on to tune the pore size of the membranes.
• The data would allow other researchers to identify the key parameters that need to be controlled in the fabrication of clay-zeolite composite membranes.

## 1 Data

### 1.1 Chemical composition of the clay

Clays are very versatile materials that are widely available in nature and possess properties not available in in pure materials that are used in the fabrication of ceramic membranes, such as: silica, alumina and zirconia (Karaborni et al., 1996; Oun et al., 2017; Hollanders et al., 2016; de Oliveira Henriques et al., 2017) [2], [3], [4], [5]. The clay used in this study was fully characterized, data for characterization are now discussed. The chemical analysis of the natural clay was performed by X-ray fluorescence (ARL-8660 X-Ray Fluorescence Spectrometer). This analysis shows that the clay consists mainly of silica SiO_2_, calcium oxide CaO, alumina Al_2_O_3_ (73%), the low presence of other alkaline and alkaline earth oxides (MgO, K_2_O Table 1).

**Table 1:** Percentages of the oxides composing the clay used.

Oxides	SiO_2_	Al_2_O_3_	Fe_2_O_3_	CaO	MgO	SO_3_	K_2_O
%	47.17	10.88	4.98	14.62	2.80	0.84	1.39

### 1.2 X-ray diffraction

**Fig.-1:**
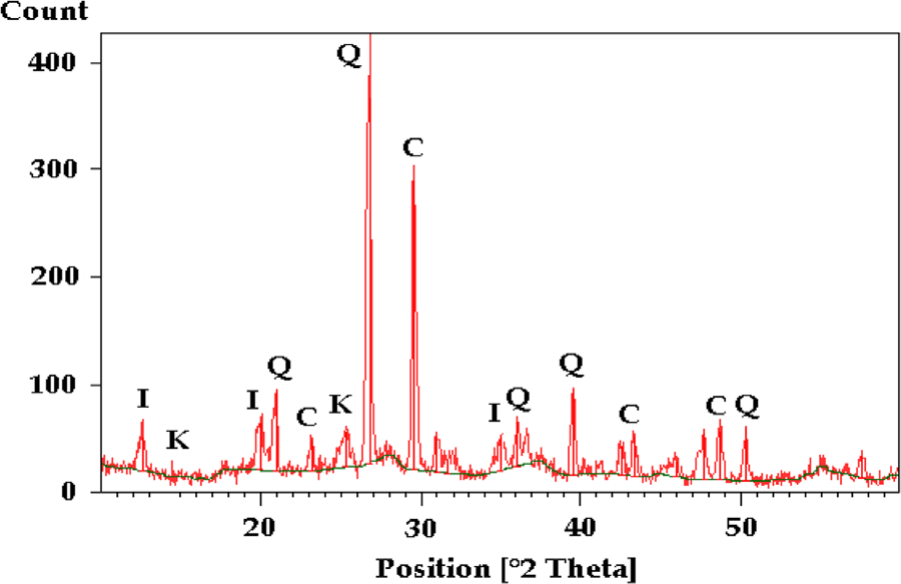
X-ray diffraction of the clay used. Q: Quartz, C: Calcite, I: Illite and K: Kaolinite.

The analysis of the clay powder fractions revealed a strong presence of silica in the form of quartz, calcite, kaolinite and illite. Fig. 1 represents the X-ray diffraction of the clay used in this study.

### 1.3 Infrared spectroscopy

**Fig. 2:**
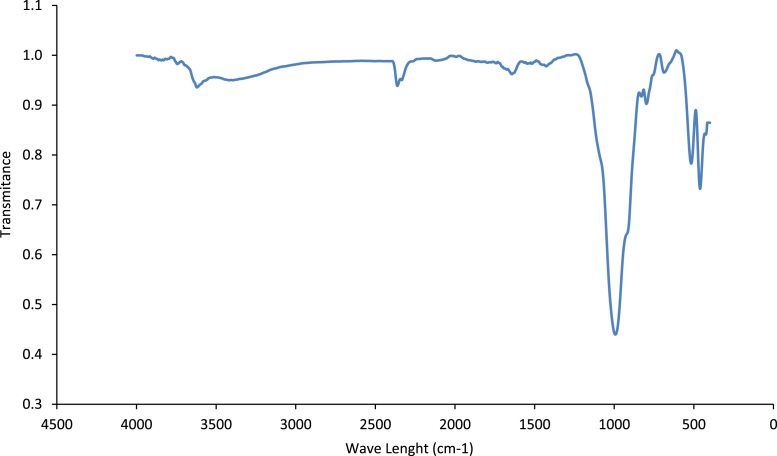
FTIR spectrum of the natural clay used.

The FTIR analysis was carried out in the spectral range (2000–4000) cm^−1^ by a Bruker Platinum ATR tensor II spectrometer with a resolution of 4 cm^−1^. Fig. 2 represents the FTIR spectrum of natural clay and different vibrations attribution of the clay are represented in Table 2.

**Table 2:** Infrared vibrations for the clay studied at two different temperatures (crude and calcined to 900 °C).

IR frequencies of crude clay	IR frequencies of calcined clay	Attributions
3711 s	–	ν Si-OH
3633 s	–	ν Al-OH
3555,5 m	3566.6 m	ν (Al,Fe)-OH
3477.75 w	3488.75 F	ν OH(OH_2_)stru
3422.2 vs	3433.3 TF	ν OH(OH_2_)stru
3275 s	3275 e	ν OH(OH_2_)hyd
1639 w	1639 F	δ OH(OH_2_)stru
1623 m	1623 m	δ OH(OH_2_)hyd
1433 m	–	ν CO_3_
1155.55 s	1090 e	
1088.7 s	1066 m	
1027.7 vs	1039 F	δ( Al.Si.Mg.Ca)OH
	1023 F	δ(AlSiMgCa)O
	974.5	Mullite δ Al_2_O
	947.5 F	
	916 F	
905.5 s		δ(CO_3_+AlOHFe(Mg))
870.2 m	–	
794.5 m	794.5 m	Quartz+H_2_O loss
777.75 m	777.75 m	
722.25 m	722.25 m	
677.8 m	677.25 m	
616.5 s	616.5 f	
566.65 w	566.5 f	
474.5 vs	474.3 TF	
444.5 w	444.5 f	
422.2 w	422.2 f	

vs: very strong, w: weak, m:medium, s:shoulder, stru:structural, hyd: hydration, ν: stretching vibration, δ: bending vibration.

### 1.4 Thermogravimetric analysis

TGA shows three weight losses during calcination.

• The first starts at 23 °C and ends at 89 °C, corresponding to the departure of the water adsorbed by the clay.
• The second starts around 400 °C and ends at 557 °C, due to the decomposition of kaolinite.
• The third begins at 639 °C and ends at 736 °C, due to the decomposition of carbonates.

#### 1.4.1 Differential thermal analysis

**Fig. 3:**
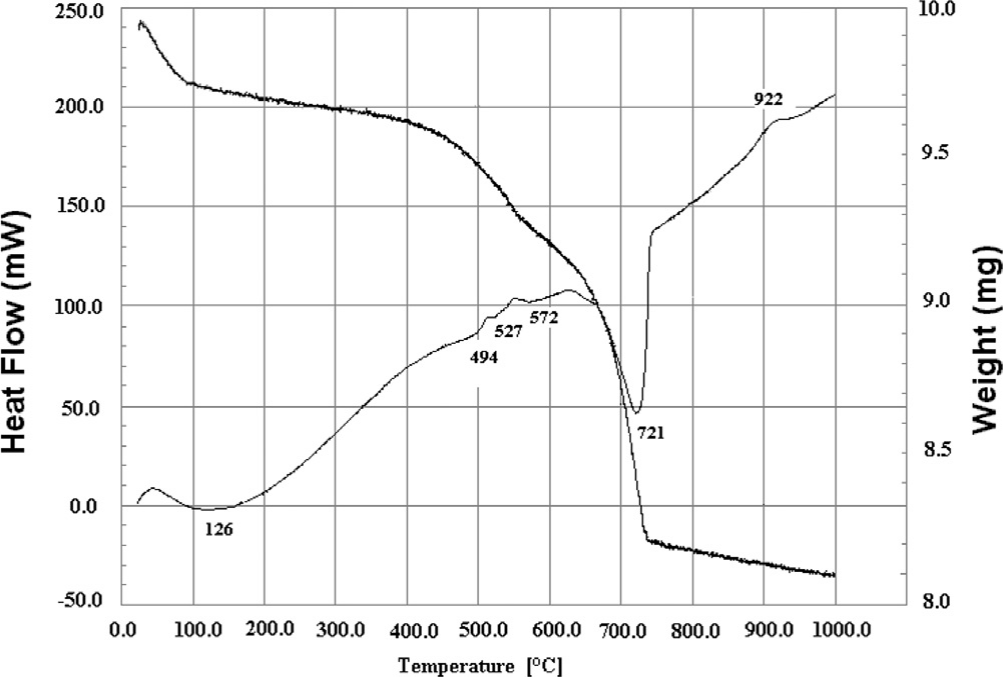
Thermal analysis (TGA and DTA) of the natural clay used.

DTA shows the presence of 5 endothermic peaks and an exothermic peak around 922 °C. TGA and DTA curves are given in Fig. 3.

#### 1.4.2 Nitrogen sorption of membrane supports

**Fig. 4:**
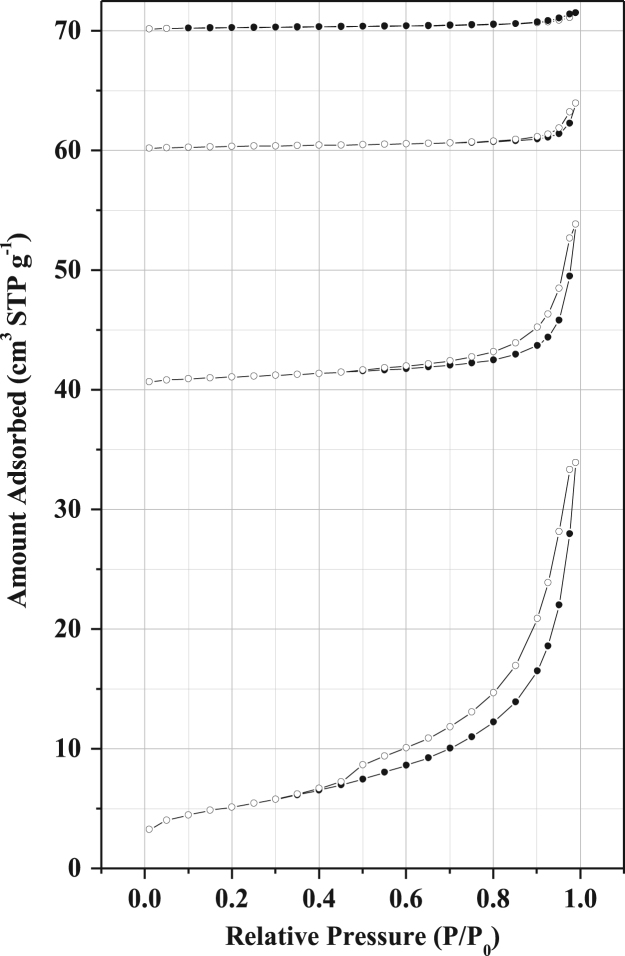
Nitrogen sorption of membrane supports made from clay only for samples sintered at 700, 750, 800 and 950 °C respectively. The isotherms were shifted vertically by 40, 60, and 70 cm^3^ STP g^−1^.

**Fig. 5:**
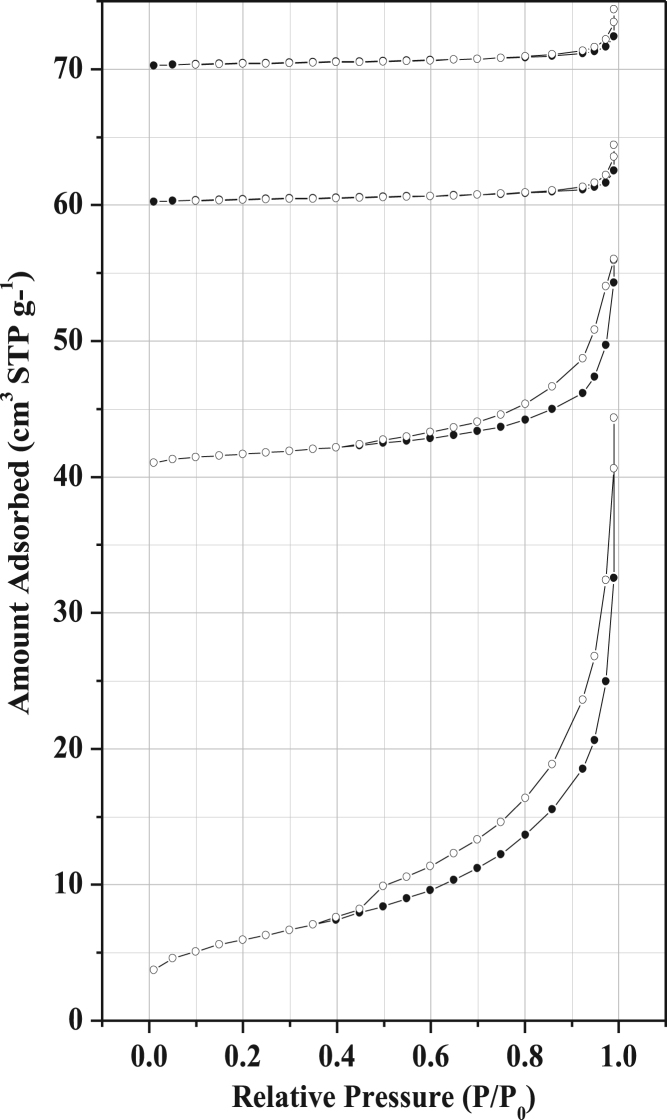
Nitrogen sorption of membrane supports made from clay and 5% activated carbon for samples sintered at 700, 750, 800 and 950 °C respectively. The isotherms were shifted vertically by 40, 60, and 70 cm^3^ STP g^−1^.

**Fig. 6:**
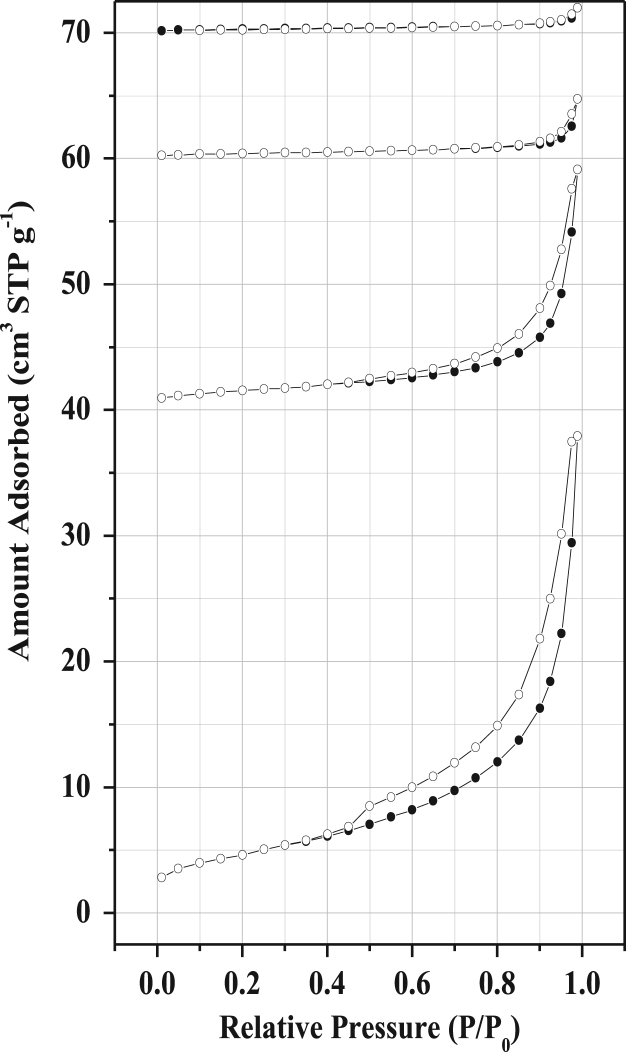
Nitrogen sorption of membrane supports made from clay and 20% starch for samples sintered at 700, 750, 800 and 950 °C respectively. The isotherms were shifted vertically by 40, 60, and 70 cm^3^ STP g^−1^.

Fig. 4, Fig. 5, Fig. 6 represent hysteresis [6], [7], [8], [9] of clay without additive, clay with 5% activated carbon and clay with 20% starch supports respectively from which different adsorption data was derived (Porosity, mesoporosity, specific surface area, volumes of the pores, volumes of mesopores, diameters of the pores).

## 2 Experimental design, materials, and methods

### 2.1 Clay supports fabrication

Homogeneous powder consisting of granulated natural clay (size between 250 and 315 µm) and well-determined percentages of organic additives undergoes a uni-axial pressure of up to 8 t. Pellets (flat supports) were obtained, these were maintained under a heat program to a final sintering temperature of 950 °C and a final sintering time of 3 h. Three substrates were prepared, respectively made from clay only, mixture of clay and 5% of activated carbon and mixture of 20% starch.

### 2.2 Silicalite membranes synthesis

**Fig. 7:**
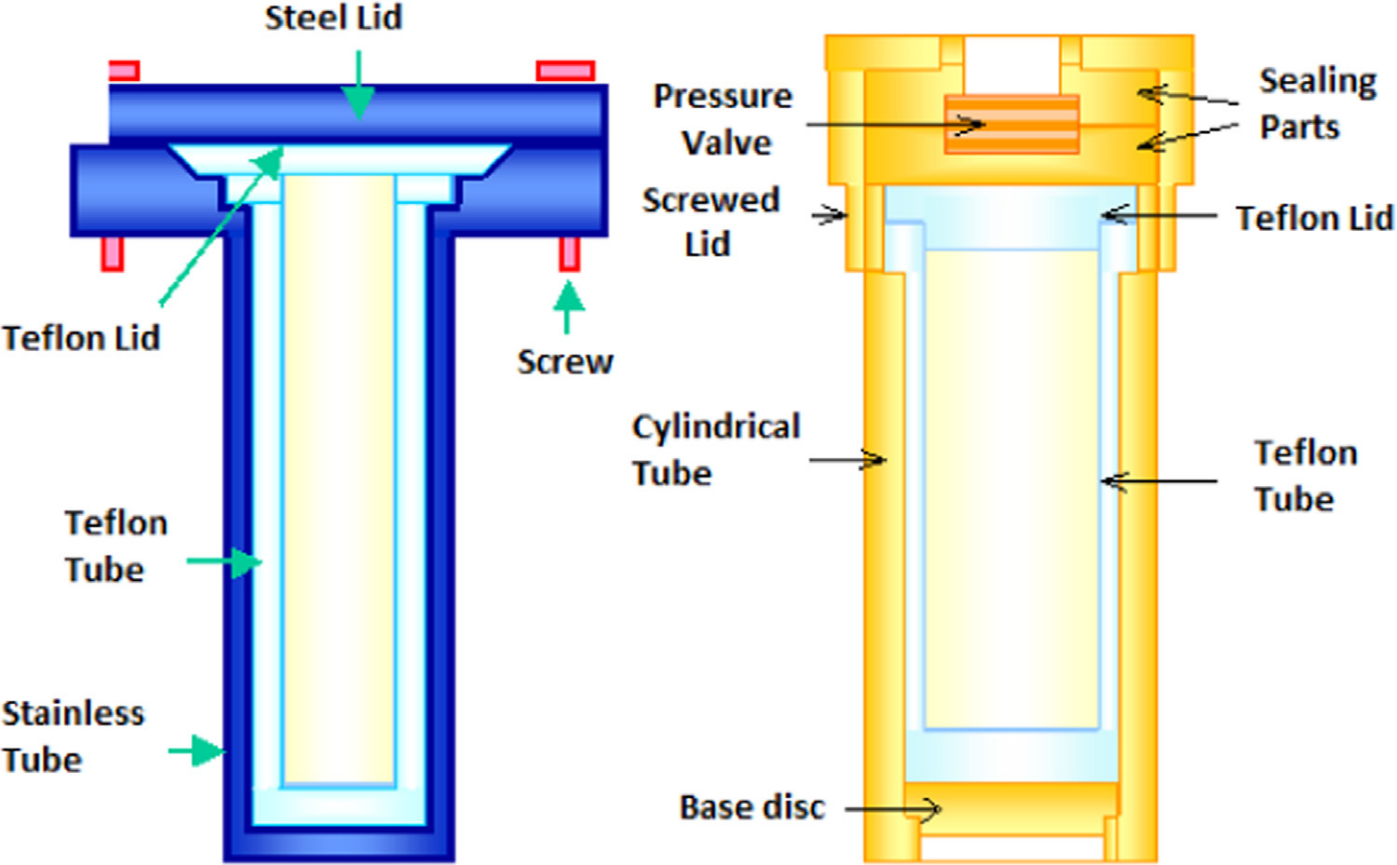
Autoclave used for the synthesis of silicalite membranes on clay supports.

A precursor gel of silicalite was prepared by mixing TEOS as silica source, tetra-n-propylammonium bromide (TPAOBr) as template and KOH as base in addition to de-ionized water. The molar composition was: 1000. H_2_O: 4.5 SiO_2_: 1.0 KOH: 1.0 TPABr. The support flat disc was introduced vertically, then the gel was poured into the Teflon lined tube and autoclave represented in Fig. 7. The autoclave was kept in an oven at 175 °C for 24 h.

#### 2.2.1 Permeation tests

**Fig. 8:**
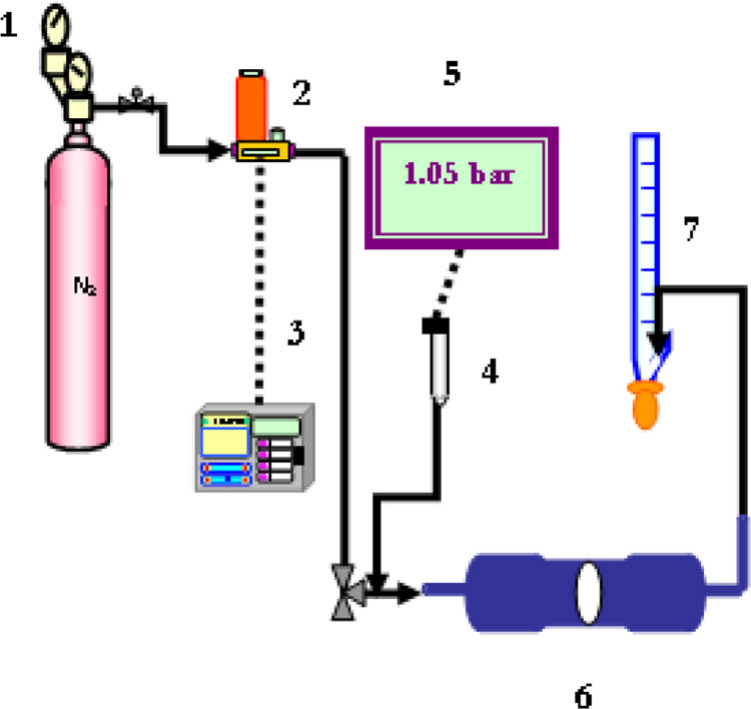
Schematic representation for the permeation measurements. 1: nitrogen cylinder (N2); 2: gas flow meter; 3: gas flow controller; 4: pressure controller; 5: pressure indicator; 6: permeation module; 7: glass flow meter.

**Fig. 9:**
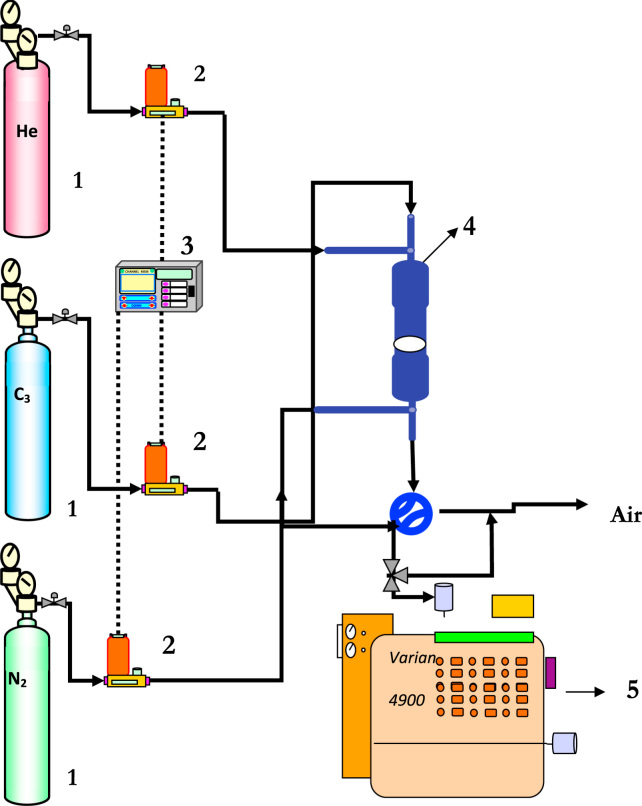
Schematic representation for the selectivity measurements. 1: gas cylinder; 2: gas flow meter; 3: gas flow controller; 4: membrane module; 5: gas chromatography machine.

A specific unit was designed and it is represented in Fig. 8 for nitrogen permeations (single gas permeation) while for the selectivity test of silicalite membranes towards N_2_, SF_6_ and propane another unit was used it is represented in Fig. 9.

### 2.3 Permeability of pure gases

Permeability is a very important feature that helps in deciding about the quality of a membrane. Measurement of permeability of N_2_, SF_6_, propane helps assuming the Knudsen and laminar contribution of a membrane.

The difference between the pores of the MFI membrane (0.55 nm) and the kinetic diameters of the N_2_ (0.364 nm), SF_6_ (0.55 nm) and propane (0.42 nm) [10] gases can cause selectivity. Selectivity is defined as the ratio of nitrogen permeability and the permeability of other gases the permeability formula given in Eq. (1).

The average pressure (*P*_av_) which represent the pressure across the membrane is equal to *P*_av_ = (2*P_at_ + Δ*P*)/2 (bar), where Δ*P* is the pressure drop of the gas across the membrane and P_at_ is the atmospheric pressure, that was found to be equal to 1.013 bar at experiment׳s time. Permeation in mol/Pa s m^2^, is defined in Eq. (1).

(1) $$Per=\frac{F\left(\frac{P_{work}\times T_{ref}}{P_{ref}\times T_{work}}\right)}{\left(\pi \times \rho^{2}\times \Delta P\times 1.344\times 10^{7}\right)}$$

*F*: Gas volumetric flow rate that is passing through the filtration area in mL/min, measured in the experiment conditions.
*T*_work_ and *P*_work_ are working temperature and pressure respectively.
*T*_ref_, *P*_ref_ are the reference temperature and pressure that are equal to 273 K and 1.0 bar respectively.
Δ*P*: Pressure difference between the inlet and the outlet of the filtration area in bar.
*ρ*: radius of the filtration area in cm.

A regular non-absorbable gas Permeation flux *F* was found to be proportional to the average pressure of the experiment and follows expression defined in Eq. (2).

(2) $$F=\frac{2}{3}\times r\times \left(\frac{\varepsilon }{\tau }\right)\times \sqrt{\frac{8}{\pi .M.R.T}}\;+\;\frac{1}{8}\left(\frac{r^{2}}{\mu .R.T}\right)\times \left(\frac{\varepsilon }{\tau }\right)\;\times P_{\mathrm{av}}$$

*r*: d/2 radius of the porous medium.
*ε*: medium porosity (dimensionless).
*ι*: tortuosity (dimensionless).
*M*: molecular weight of gas (kg/mol).
*T*: temperature (K).
*μ*: gas viscosity (Pas).
*R*: gas constant (*R* = 8.314 J/mol K).

Substituting each parameter by its value and holding constant values, Eq. (2) becomes,

(3) $$F=\;\alpha \;+\;\beta \times P_{\mathrm{av}}$$

where *α* and *β* are Knudsen laminar and viscous coefficient respectively.

Knudsen percentage contribution is defined as the ratio of the coefficient α and the total permeation flux at a pressure of 1.0 bar.

(4) $$\%Knudsen=\frac{\alpha }{\alpha \;+\;\beta }\times 100$$

**Fig. 10:**
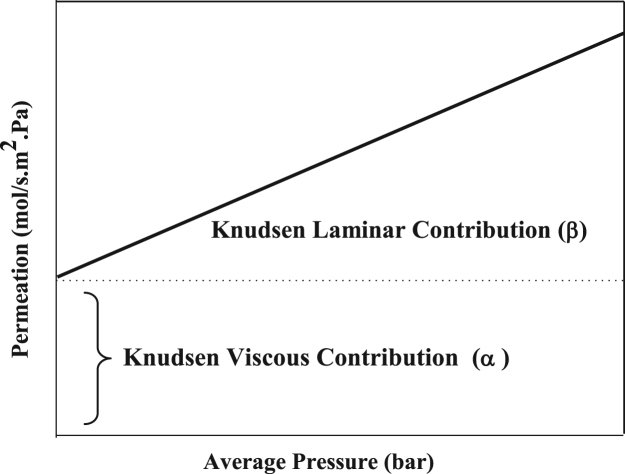
Theoretical laminar and viscous Knudsen contributions obtained from the computational values of the permeation as a function of the average pressure.

**Fig. 11:**
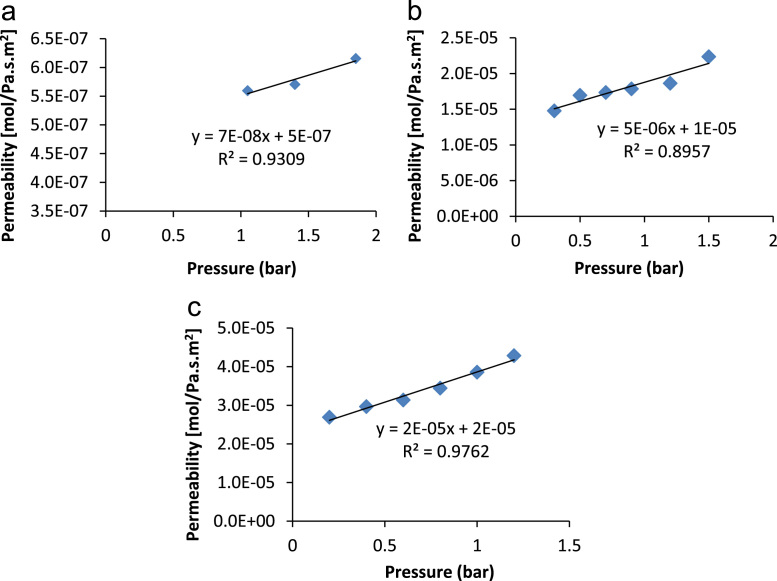
Charts for supports permeations used for the determination of Knudsen and Laminar contribution; (**a**) support made from clay without additive; (**b**) support made from clay and 5% activated carbon; (**c**) support made from clay mixed with 20% starch.

**Fig. 12:**
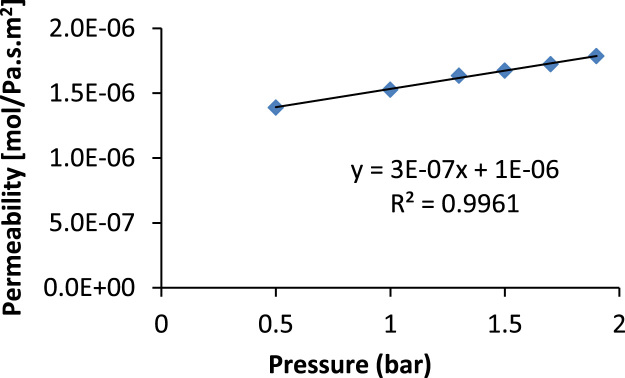
Permeation Chart for zeolite membrane deposited on activated carbon-clay support used for the determination of Knudsen and Laminar contribution; deposition at 90 °C during 4 h.

**Fig. 13:**
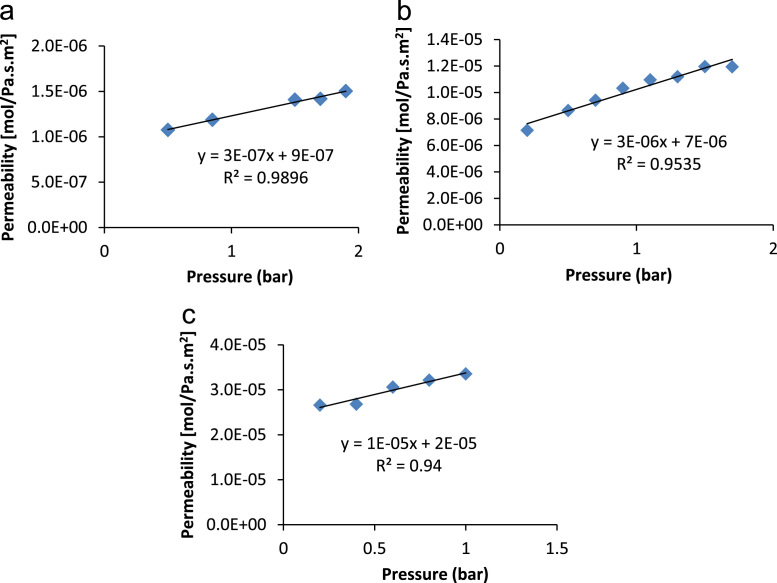
Permeation Charts for zeolite membranes deposited on starch-clay supports used for the determination of Knudsen and Laminar contribution; (**a**) deposition at 90 °C during 6 h; (**b**) deposition at 170 °C during 24 h; (**c**) deposition at 170 °C during 25 h.

Eq. (4) is of great practical use; it is used to describe the transport in the gas phase of a non-absorbable gas across a porous medium. If permeation flux defined in Eq. (3) is plotted against the average pressure a linear graph is obtained and it is illustrated in Fig. 10, where the intercept corresponds to the Knudsen viscous contribution (*α*) and the slope to the laminar Knudsen coefficient (*β*). These parameters were determined for clay supports as well as clay-zeolite composite membranes and they are represented in Fig. 11, Fig. 12, Fig. 13.
