# Supplementary material for: Dataset in the production of composite clay-zeolite membranes made from naturally occurring clay minerals
Source: Data Brief. 2018 Jul 6;19:2267–78. doi: 10.1016/j.dib.2018.06.117 (PMC6141442; doi:10.1016/j.dib.2018.06.117)
Supplement: Supplementary file 1 — Transparency document [file mmc1.docx]

**Cover letter**

Dear Editor,

Please find enclosed our data manuscript, “Dataset on the production of composite clay-zeolite membranes from naturally occurring clay minerals” By Abdelaziz Elgamouz et al., which we would like to submit for publication as a data article in the Journal of Data in Brief.

To our knowledge, this is the first data report showing techniques and methods used in the fabrication of clay-zeolite composite, that should be very useful for other scientists during the design of ceramic membranes. We believe our findings would appeal to the readership of the Journal of Data in Brief.

We confirm that this data has not been published elsewhere and is not under consideration by any other journal. We declare also that that there is no conflict with any parties as far as this data is concerned, all funding bodies were mentioned in the data paper.

All authors have approved the data and agree with its submission to the Journal of Data in Brief.

Please address all correspondence to: Dr. Abdelaziz Elgamouz, Department of Chemistry, College of Sciences, P.O. Box 27272, Sharjah, UAE. (aelgamouz@sharjah.ac.ae)

We look forward to hearing from you at your earliest convenience.

Abdelaziz ELGAMOUZ PhD, DESA, Bsc

Assistant Professor in Chemistry

Chemistry Laboratories’ Coordinator

Department of Chemistry

College of Sciences

P.O. Box 27272

Sharjah, UAE

Phone: +971 (0) 65166 769 (Direct line) Ext: 1769

Fax: +971 6 505 1769
